# Ethnic differences in COVID-19 deaths across various waves of Coronavirus pandemic in Netherlands: Felix Chilunga

**DOI:** 10.1093/eurpub/ckac129.604

**Published:** 2022-10-25

**Authors:** FP Chilunga, L Stoeldraier, C Agyemang, K Stronks, H Harmsen, AE Kunst

**Affiliations:** 1 Public and Occupational Health, Amsterdam UMC, Amsterdam, Netherlands; 2 Demography Group, Central Bureau of Statistics, Den Haag, Netherlands

## Abstract

**Background:**

It is not known how ethnic differences in COVID-19 deaths in the Netherlands evolved throughout the pandemic, especially after introduction of ethnicity-oriented COVID-19 prevention measures. We investigated associations between ethnicity and COVID-19 deaths across first wave of the pandemic, inter-wave period, and second wave in the Netherlands.

**Methods:**

We obtained multiple registry data from Statistics Netherlands spanning from 01 March 2020 to 14 March 2021 comprising of 17.4 million inhabitants. We estimated incidence rate ratios (IRRs) for COVID-19 deaths among ethnic groups using Poisson regression models and adjusted for relevant socio-demographic factors. We used similar models to estimate IRRs for non-COVID-19 deaths among ethnic groups.

**Results:**

Ethnic minority populations exhibited higher risk of COVID-19 deaths than the Dutch origin population throughout various study periods. The most elevated risk of COVID-19 deaths was in populations originating from low- and middle-income countries, especially those with Turkish, Moroccan, and Surinamese background. The elevated risk of COVID-19 deaths among ethnic minority groups (as compared to Dutch origin population) was higher in inter-wave period (4 times higher) and second wave (2 times higher) when compared to the first wave (1.5 times as higher). Ethnic differences in COVID-19 deaths were larger compared to non-COVID-19 deaths.

**Conclusions:**

Ethnic differences in COVID-19 deaths persisted across first wave, inter-wave period and second wave in the Netherlands despite introduction of ethnicity-oriented prevention measures. Research on explanatory mechanisms and novel prevention measures are needed to address the ongoing differences in COVID-19 deaths across ethnic groups.

**Key messages:**

• Ethnic differences in COVID-19 deaths persisted in the Netherlands despite introduction of ethnicity-oriented prevention measures.We therefore call for better prevention measures.

• Well known drivers of SARS-CoV-2 infection such as household wealth, did not explain our findings calling for an in-depth understanding of drivers of ethnic differences in COVID-19 deaths.

